# Selective involution of thymic medulla by cyclosporine A with a decrease of mature thymic epithelia, XCR1^+^ dendritic cells, and epithelium-free areas containing Foxp3^+^ thymic regulatory T cells

**DOI:** 10.1007/s00418-021-01993-y

**Published:** 2021-05-16

**Authors:** Yasushi Sawanobori, Yusuke Kitazawa, Hisashi Ueta, Kenjiro Matsuno, Nobuko Tokuda

**Affiliations:** Department of Anatomy, School of Medicine, Dokkyo Medical University, Tochigi, Japan

**Keywords:** Immunosuppressive drug, Thymus, Dendritic cells, Thymic epithelial cells, Regulatory T cells, Thymic structure

## Abstract

Immunosuppressive drugs such as cyclosporine A (CSA) can disrupt thymic structure and functions, ultimately inducing syngeneic/autologous graft-versus-host disease together with involuted medullas. To elucidate the effects of CSA on the thymus more precisely, we analyzed the effects of CSA on the thymus and T cell system using rats. In addition to confirming the phenomena already reported, we newly found that the proportion of recent thymic emigrants also greatly decreased, suggesting impaired supply. Immunohistologically, the medullary thymic epithelial cells (mTECs) presented with a relative decrease in the subset with a competent phenotype and downregulation of class II major histocompatibility complex molecules. In control rats, thymic dendritic cells (DCs) comprised two subsets, XCR1^+^SIRP1α^−^CD4^−^ and XCR1^−^SIRP1α^+^CD4^+^. The former had a tendency to selectively localize in the previously-reported epithelium-containing areas of the rat medullas, and the number was significantly reduced by CSA treatment. The epithelium-free areas, another unique domains in the rat medullas, contained significantly more Foxp3^+^ thymic Tregs. With CSA treatment, the epithelium-free areas presented strong involution, and the number and distribution of Tregs in the medulla were greatly reduced. These results suggest that CSA inhibits the production of single-positive thymocytes, including Tregs, and disturbs the microenvironment of the thymic medulla, with a decrease of the competent mTECs and disorganization of epithelium-free areas and DC subsets, leading to a generation of autoreactive T cells with selective medullary involution.

**Supplementary Information:**

The online version contains supplementary material available at 10.1007/s00418-021-01993-y.

## Introduction

Graft-versus-host disease (GVHD) is a common complication accompanying allogeneic hematopoietic stem cell transplantation and is caused primarily by differences in the major histocompatibility complex (MHC) and/or minor antigens. However, even after transplantation of autologous stem cells, GVHD-like syndrome can occur, called engraftment syndrome or autologous GVHD (Cornell et al. 2015; Kline et al. 2008). Animal models of this syndrome, induced by a combination of hematopoietic stem cell transplantation followed by 3–4 weeks of cyclosporine A (CSA) administration and ~ 3 weeks of CSA-free period after withdrawal have been reported in rats and mice (Cheney and Sprent 1985; Glazier et al. 1983), and called syngeneic GVHD. In these long-term induction models, the thymus has been suggested to be involved in the development of the syndrome because thymectomized animals fail to exhibit the disease (Sorokin et al. 1986). Furthermore, short-term CSA administration can also induce autoreactive T cells within 4 days in the thymus, and 7 days in the lymph nodes (LNs), as assessed by the local popliteal LN swelling after injection of the cells into the footpads of recipient animals (Wu and Goldschneider 1999, 2001). These T cells were considered to be involved in the pathogenesis of autologous GVHD and, thus, this short-term protocol may be useful for analyzing the early effects of CSA treatment leading to the manifestation of the disease. Therefore, we deployed this model in this study.

Concerning abnormalities in thymi and T cell generation after CSA administration, there have been many reports demonstrating involution of the thymic medulla (Schuurman et al. 1990), decreased single-positive thymocytes and peripheral T cells (Kosugi et al. 1989), downregulated expression of class II MHC molecules (MHCII) on medullary thymic epithelial cells (mTECs) (Fletcher et al. 2009), suppressed regulatory T cell (Treg) generation (Coenen et al. 2007), and the generation of autoreactive T cells (Wu and Goldschneider 1999, 2001). However, these reports mostly studied isolated cells, and in situ distribution of TECs, thymic dendritic cell (tDC) subsets, and thymic Tregs (tTregs) after CSA treatment have not yet been studied. To perform positive or negative selection, cortical TECs (cTECs) and mTECs, and medullary tDCs present MHC molecules and antigens (Klein et al. 2014; Wang et al. 2019). However, the effects of CSA on these antigen-presenting cells are still unclear.

Previously, using mAbs ED18 and ED21, we found two novel subsets of TECs, mTEC1 (ED18^+^ED21^−^) and mTEC2 (ED18^+^ED21^+^). mTEC1 is more competent with abundant expression of functional molecules, such as AIRE and MHCII (Sawanobori et al. 2014). This subset may be affected by CSA. In contrast to the medullary areas containing mTECs (medullary epithelium-containing areas, mECAs), rat thymi have unique areas lacking mTECs (medullary epithelium-free areas, mEFAs). The properties and roles of these areas in normal and CSA-affected states are not yet known.

In this study, we investigated the effects of short-term CSA treatment on thymocytes and T cell subsets in the thymus and LNs and the autoreactivity of the peripheral T cell pool. We focused on the immunohistological analysis of the thymic medulla, in regards to the in situ distribution of mECAs and mEFAs, mTEC subsets, tDC subsets, and Tregs.

## Materials and methods

### Animals

Eight-weeks old inbred male Lewis rats were purchased from SLC Co. (Shizuoka, Japan). The administration of CSA solution or control solvent was started at 8 weeks of age. Normal (untreated) thymi were collected at 8 or 9 weeks of age. All rats were reared under specific pathogen-free conditions. Animal handling and care protocols were approved by Dokkyo Medical University’s Regulations for Animal Experiments and with Japanese Governmental Law (No. 105).

### Antibodies

The antibodies used for immunohistology and flow cytometric analysis are listed in Table 1. Some antibodies were purified from culture supernatants and conjugated in-house.

**Table 1:** Antibodies

Antigen	Isotype	Clone	Conjugate	Source
Unknown	Mouse IgM	ED18	Biotin^a^, Alexa Fluor 594^b^	Produced at Neuroscience Campus Amsterdam (the Netherland) ^c^
Unknown	Mouse IgM	ED19	Biotin^a^	Produced at Neuroscience Campus Amsterdam (the Netherland) ^c^
Unknown	Mouse IgM	ED21	Unconjugated, biotin^a^, Alexa Fluor 488^b^	Produced at Neuroscience Campus Amsterdam (the Netherland) ^c^
CD4	Mouse IgG1	W3/25	FITC, PerCP-Cy5.5, phycoerythrin (PE)	BioLegend (San Diego, CA, USA)
CD8α	Mouse IgG1	OX8	FITC, PE	BioLegend
CD11b/c	Mouse IgG2a	OX42	PE	Biolegend
CD25	Mouse IgG1	OX39	FITC, PE	BioLegend
CD45R	Mouse IgG2b	HIS24	PE	eBioscience (Waltham, MA, USA)
CD80	Mouse IgG1	3H5	PE	BioLegend
CD86	Mouse IgG1	24F	PE	BD Bioscience (Franklin Lakes, NJ, USA)
CD90	Mouse IgG1	OX7	PE, PerCP-Cy5.5	BioLegend
CD103	Mouse IgG1	OX62	Unconjugated, FITC	ECACC^c, d^,BioLegend
CD172a (SIRP1α)	Mouse IgG2a	OX41	PE	BioLegend
CD205	Mouse IgG1	HD83	PE	BioLegend
Foxp3	Rat IgG2a	FJK-16 s	Biotin, eFluor660	eBioscience
Helios	Armenian hamster IgG	22F6	PE	BioLegend
MHC II	Mouse IgG1	OX6	Unconjugated, Alexa Fluor 647^b^	AbD Serotec, CEDARLANE (Ontario, Canada)
MHC II (RT1B^l^)	Mouse IgG1	OX3	Alexa Fluor 647^b^	ECACC^c, d^
TCRαβ	Mouse IgG1	R73	Alexa Fluor 647^b^	ECACC^c, d^
XCR1	Mouse IgG2b	ZET	Unconjugated, biotin	BioLegend
Type IV collagen	Rabbit IgG	Polyclonal	Unconjugated,	Cosmo Bio, LSL (Tokyo, Japan)
Isotype control	Mouse IgG	Polyclonal	Unconjugated,	Jackson ImmunoResearch (West Grove, PA, USA)
Isotype control	Mouse IgG1	MOPC-21	Alexa Fluor 647	BioLegend
Isotype control	Mouse IgG2b	MPC-11	Unconjugated, PE	BioLegend
Isotype control	Mouse IgM	11E10	Biotin^a^, Alexa Fluor 488^b^ Alexa Fluor 594^b^	eBioscience
Isotype control	Rat IgG2a	RTK2758	Biotin	BioLegend
Anti-mouse IgG	Goat IgG	Polyclonal	Alkaline phosphatase	Sigma-Aldrich (Saint Louis, MO, USA)
Anti-mouse IgG	Horse IgG	Polyclonal	Biotin	Vector Laboratories (Burlingame, CA, USA)
Anti-mouse IgM	Donkey IgG	Polyclonal	Alkaline phosphatase	Jackson ImmunoResearch
Anti-rabbit IgG	Goat F(ab’)_2_	Polyclonal	Peroxidase	Jackson ImmunoResearch
Anti-biotin	Goat IgG	Polyclonal	Alkaline phosphatase	Sigma-Aldrich
Streptavidin			Alexa Fluor 594, PerCP-Cy5.5	Invitrogen (Waltham, MA, USA)

^a^Conjugated in our facility using the Biotin Labeling Kit—NH2 (Dojindo Molecular Technologies, Kumamoto, Japan)

^b^Conjugated in our facility using the Alexa Fluor® conjugate kit (Thermo Fisher)

^c^Hybridomas were cultured and the produced antibodies were purified in our facility

^d^The European Collection of Authenticated Cell Cultures (Salisbury, UK)

### CSA administration

CSA and olive oil were purchased from FUJIFILM Wako Pure Chemical (Osaka, Japan). CSA was dissolved into olive oil at a concentration of 30 mg/ml in a 65 °C water bath with stirring (Sorokin et al. 1986). This solution was stored at − 20 °C until administration. A total of 100 μl of the CSA solution per 200 g of body weight was administered subcutaneously into rats, for a final dosage at 15 mg/kg/day (Wu and Goldschneider 1999). The control group received solvent only. Rats were administrated CSA solution or solvent for 16 consecutive days. The next day of final administration, rats were sacrificed and analyzed.

### Histology and immunohistology

Freshly frozen samples were sectioned at 4 μm thickness for general histology, or 2 μm for serial sections. For hematoxylin–eosin (H&E) staining, sections of freshly frozen tissues were air-dried for 2 h, and then hydrated in PBS. After fixation in 1% glutaraldehyde/PBS and 4% paraformaldehyde-1% calcium chloride solution, sections were stained in hematoxylin solution (Sakura Finetek Japan, Tokyo, Japan) for 5–10 min. After washing in water for 10 min, sections were stained in eosin solution (Sakura Finetek Japan) for 3 min, and then dehydrated in 70, 80, 90, and 99.5% ethanol for 1 min each, followed by clearing in xylene for 5 min. Sections were mounted with Entellan new (Merck, Kenilworth, NJ).

For immunohistology, Sections of freshly frozen tissues were fixed in acetone and immunostained as described previously (Sawanobori et al. 2014). Briefly, acetone-fixed sections were hydrated in Tris-buffered saline (pH 7.4), then fixed again in 4% paraformaldehyde-1% calcium chloride solution. After blocking with Block Ace (KAC, Kyoto, Japan), sections were incubated with antibodies. Conjugations of primary antibodies and used secondary antibodies are described in the legends of each figure.

For light microscopy, sections were colored with Vector Blue substrate (Vector Laboratories) or using the New Fuchsin Substrate System (Agilent, Santa Clara, CA, USA) after incubated with alkaline phosphatase-conjugated secondary antibodies. Then type IV collagen, which reveals the tissue framework (Matsuno et al. 1996), was stained with anti-type IV collagen serum (Cosmo Bio, LSL, Tokyo, Japan) followed by peroxidase-conjugated anti-rabbit IgG antibody and 3,3’-diaminobenzidine (DAB) substrate (Dojindo Molecular Technologies). Photomicrographs were captured with a Microphot-FX microscope using a Plan Apo objective lens series (Nikon, Tokyo, Japan) and a DP26 digital camera (Olympus, Tokyo, Japan), or a BX53 microscope with a UPlanFL N objective lens series and a DP27 (Olympus). Montage images were synthesized and area measurements were processed using cellSens software (Olympus). The original resolution of the pictures is 1224 × 960 pixels. Exposure settings were fixed for each experiment.

For fluorescent microscopy, sections were fixed in 4% paraformaldehyde-PBS and mounted with Fluorescent Mounting Media (KPL, Gaithersburg, MD, USA) after immunostaining. Multicolor fluorescence images were captured using an Axioskop 2 Plus fluorescence microscope equipped with a Plan-Apo objective lens series and an AxioCam MRm camera (Zeiss, Oberkochen, Germany). The original resolution of the pictures is 1388 × 1040 pixels. Exposure settings were fixed for each experiment. Filter Sets 17, XF406, and 32 were used to capture Alexa Fluor 488, 594, and 647 respectively.

### Fluorescent image analysis

For the analysis of immunofluorescent images, multicolor fluorescence images were captured using a BZ-9000 fluorescent microscope (Keyence, Osaka, Japan) with the CFI Plan Fluor Objective lens series (Nikon). BZ Filter GFP-BP, Texas Red, and Cy5 were used to capture Alexa Fluor 488, 594, and 647 respectively. The original resolution of the pictures is 680 × 512 pixels. Exposure settings were fixed for each antibody. The images were converted into BZ-X format and analyzed by BZ-X analyzer software (Keyence) for areas of thymic epithelial cell subsets or tDC subsets and MHCII expression. For tDCs, we estimated either XCR1^+^MHCII^+^ surface area/mm^2^ because the outline of each cell was difficult to determine. For this, the ECA/EFA ratio was calculated as XCR1^+^MHCII^+^ area/mm^2^ in ECA to those of in EFA.

### Cell isolation and flow cytometry

For flow cytometric analysis, thymi, spleens, and LNs were injected with 0.2% collagenase D (Roche Diagnostics, Indianapolis, IN, USA), 0.01% DNase I (Roche), and HBSS (37 °C, pH 7.4), and then cut into slices 1 to 2 mm-thickness and incubated in 0.1% collagenase D/0.1% DNase I/HBSS at 37 °C for 25 min. After incubation, samples were supplemented with EDTA to 2.5 mM, teased, and filtered using 50-μm nylon mesh. The digested cell suspension was centrifuged and resuspended in 15% OptiPrep (Axis-Shield, Oslo, Norway)/PBS(−) in centrifuge tubes, and 12% OptiPrep/PBS(−) and then PBS(−) overlaid on the cell suspension. The tubes were centrifuged at 600*g* for 25 min at room temperature. Cells at the interface between the 15 and 12% OptiPrep, and the 12% OptiPrep and PBS(−) were considered thymocytes/lymphocytes, and DC-containing low-density cells respectively. These cells were collected and subjected to flow cytometric analysis. Cells were stained using the conventional method. For analysis of thymocyte subsets, single-positive cells were further defined as the TCR^hi^ population, because strong inhibition of single-positive cells by CSA made contamination of double-positive or double-negative cells into single-positive gates non-negligible. For analysis of tDCs, low-density cells were further purified with anti-DC (OX62: CD103) microbeads and an autoMACS (Miltenyi Biotec, North Rhine-Westphalia, Germany). To stain Foxp3, the Foxp3/Transcription Factor Staining Buffer Set (eBioscience) was used. Stained cells were acquired using an Attune NxT flow cytometer (Thermo Fisher). Data were analyzed using FlowJo V10.5.3 (FlowJo LLC, Ashland, OR).

### Mixed leukocyte reaction

The LNs of CSA-administered or control rats and spleens of control rats were digested and cells isolated as described above. To prepare responder T cells, LN cells were suspended in GIT medium (FUJIFILM Wako Pure Chemical) and stained with PE-conjugated anti-CD45R and anti-CD11b/c antibodies at 4 °C for 15 min. Because CD25 is often exploited for the depletion of Tregs (Yamazaki et al. 2006; Mikulic et al. 2017), PE-conjugated anti-CD25 antibody was added to obtain Treg-depleted responder T cells. Cell suspensions were washed with 2 mM-EDTA/PBS(−) (MACS buffer) twice, followed by incubation with anti-PE microbeads (Miltenyi Biotech) in GIT medium at 4 °C for 15 min. Cells were washed again and subjected to the autoMACS (Miltenyi Biotech) depletion protocol to obtain purified T cells. The purity of the T cells was > 96%. Stimulator DCs were isolated from the spleen cells using Anti-DC (OX62) MicroBeads (Miltenyi Biotech) and the positive selection protocol of the AutoMACS. Isolated T cells were stained with CytoTell™ green (AAT Bioquest, Sunnyvale, CA, USA) reagent following the manufacturer’s protocol. A total of 1 × 10^5^ responder cells and 9 × 10^4^ stimulator cells were seeded into each well of 96-well flat bottomed culture plates (Bio-Rad, Hercules, CA, USA) and incubated at 37 °C, in 5% CO_2_ for 7 days. After incubation, cells were stained with Alexa Fluor 647-conjugated anti-MHCII antibody to distinguish DCs and T cells, and then counted and captured using an Attune NxT flow cytometer. To exclude dead cells, propidium iodide (PI, Dojindo Molecular Technologies) was added to samples at a final concentration of 1 μg/ml just before the capture. Proliferating cells were defined as CytoTell dull or negative cells.

### Statistical analysis

Statistical analysis was performed using the Student’s *t* test. For comparison between control and CSA-administered rats, data sets were considered as two-tailed distribution and heteroscedastic. For examination of cell distribution, data sets were considered as two-tailed distribution and paired. Error bars indicate standard deviations.

## Results

### Effects of CSA on T cell lineages of the thymus and lymph nodes

**Fig. 1:**
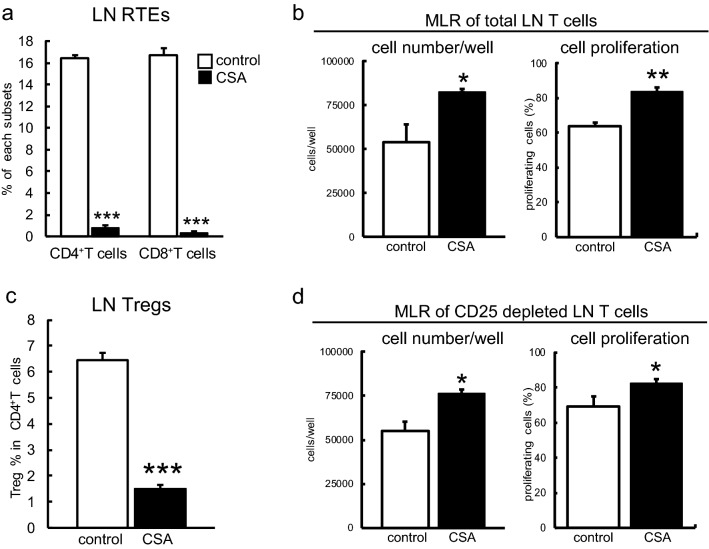
Effects of CSA on T cell homeostasis and autoreactivity. Thymi and peripheral LNs from control and CSA-administered rats were digested and analyzed by flow cytometry. **a**,** c** Ratio of CD90^+^ RTEs in peripheral LNs (**a**) and ratio of CD4^+^CD25^+^Foxp3^+^ Tregs in peripheral LNs (**c**) are displayed. Each group contains three rats in (**a**) and five rats in (**c**). **b**,** d** MLR assays were performed following the scheme in Fig. S1 to examine the autoreactivity of total peripheral T cells (**b**) and CD25-depleted T cells (**d**). Each culture was prepared in triplicate. Representative data from two independent experiments are shown. *p < 0.05, **p < 0.005, ***p < 0.0005

First, we confirmed decreased CD4 and CD8 single-positive thymocytes and increased double-positive thymocytes (data not shown), as well as a decrease of peripheral T cells as previously reported (Kosugi et al. 1989). As a new finding, recent thymic emigrants (RTEs), defined by the expression of CD90 (Hosseinzadeh and Goldschneider 1993), were strongly reduced, suggesting impaired egress and supply of newly developed T cells from the thymus (Fig. 1a).

For the detection of autoreactivity of peripheral T cells, total T cells were purified from peripheral LNs and cocultured with splenic DCs of the same strain (Fig. S1). T cells from CSA-treated LNs exhibited enhanced proliferation when assessed for the number of living T cells in each well and the ratio of proliferating cells (Fig. 1b) compared to control. For Tregs, LNs exhibited a significant decrease in CD4^+^CD25^+^Foxp3^+^ cells (Fig. 1c). To examine the presence of autoreactivity of conventional LN T cells per se, we depleted CD25^+^ cells from the responders and compared the reactivity between the CSA and control groups. CD25^−^ T cells from CSA-administered rats still exhibited enhanced reactivity compared to control CD25^−^ T cells (Fig. 1d), indicating that conventional T cells in CSA rats comprise significantly more autoreactive cells than control rats. Collectively, these data indicate that our CSA protocol essentially reproduced results reported by other laboratories (Wu and Goldschneider 1999, 2001) in another experimental system, and that it may lead to autologous GVHD. A new finding was the stronger reduction in RTEs than peripheral T cells. We also observed that CD25^−^ conventional T cells in CSA-administered rats contained significantly more autoreactive cells than control CD25^−^ T cells. In auto MLR, it was detectable without Treg involvement.

### Immunohistological examination of thymic abnormalities induced by CSA treatment

**Fig. 2:**
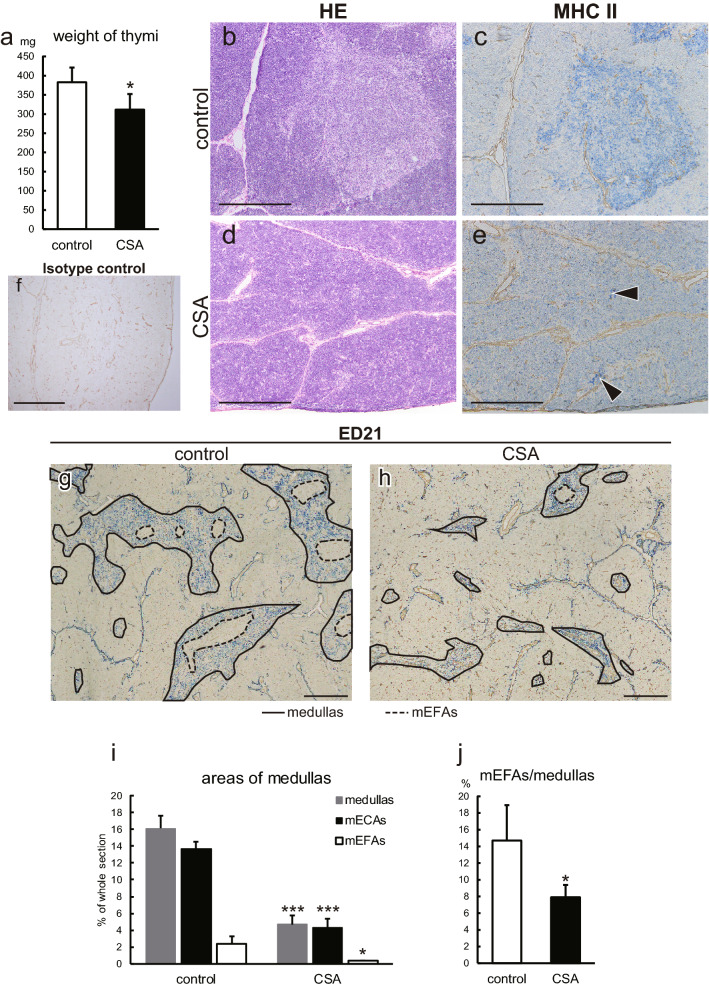
Histological overview of CSA-treated thymi. **a** Weight of thymi. **b**–**f** Sections of freshly frozen thymi from control (**b**, **c**, **f**) and CSA (**d**, **e**) rats were H&E (**b**, **d**) and immunohistologically stained with an anti-MHCII (**c**, **e**) or polyclonal mouse IgG isotype control (**f**) antibodies followed by alkaline phosphatase-conjugated anti-mouse IgG antibody. **g**,** h** Freshly frozen sections of control (**g**) and CSA (**h**) rats were immunostained with biotin-conjugated ED21 antibody followed by alkaline phosphatase-conjugated anti-biotin antibody. Medullary areas and mEFAs are indicated by solid and broken lines, respectively. **i**,** j** Ratios of medullary, mECA, and mEFA areas in total thymic cross-sectional areas (**i**) and ratios of mEFA areas in the medullas (**j**) were measured and calculated from ED21-stained sections. On immunohistological sections, type IV collagen was also stained. Photomicrographs were taken with a 4x (**g**, **h**) or 10x (**b**–**f**) objective lens. Montage images were synthesized to obtain low-powered images (**g**, **h**). Scale bars indicate 500 μm. Data from five rats in each group were statistically analyzed (**a**, **i**, **j**). *p < 0.05, ***p < 0.0005

Next, we examined control and CSA-treated thymi to understand the overall structures using H&E and immunohistological staining of fresh-frozen sections. With CSA treatment, thymic weight slightly, but significantly, decreased (Fig. 2a). In the control thymus, medullas were clearly identifiable with H&E staining (Fig. 2b) and anti-MHCII immunostaining (Fig. 2c), appearing as pale thymocyte-sparse areas and highly MHCII-expressing cells (presumably mTECs and tDCs), respectively. As we reported previously, ED21 could depict mECAs and mEFAs within medullas (Fig. 2g).

With CSA treatment, the thymic medulla was involuted and the boundaries of the cortexes and medullas became rather obscure on the H&E-stained sections (Fig. 2d). Besides, MHCII expression was not homogeneous in the involuted medullas and presented spotty patterns (Fig. 2e), making discrimination of the medulla from the cortex difficult. To depict the medulla precisely, We compared several markers. As we demonstrated in our previous paper (Sawanobori et al. 2014), anti-keratin 5 (K5) antibody and *Ulex europaeus* lectin 1 (UEA-1), which are commonly used to identify thymic medulla of mice, also stained cTECs in the rat thymus (Fig. S2a, b). ED18, the antibody that we used to identify mTEC1 in combination with ED21, also stained cTECs (Fig. 2c). On the other hand, ED21 could depict rat medullas consistently even in the cases of CSA-treated thymi (Fig. S2d, Fig. 2h). Accordingly, we deployed ED21 to assess the proportions of medullas, mECAs, and mEFAs. The medullary areas of CSA-treated thymi decreased to one-third of the control, with a decrease in both mECAs and mEFAs (Fig. 2g–i). In particular, the decrease in mEFAs was more profound (Fig. 2j), while immunofluorescent staining did not reveal a skewed distribution of single-positive thymocytes in mEFAs and mECAs (data not shown). However, in the thymic cortex, the number of cTECs detected with ED19 antibody and their MHCII expression seemed unchanged (Fig. S3).

### Impairment of the competent mTEC1 subset in the CSA-treated thymus

**Fig. 3:**
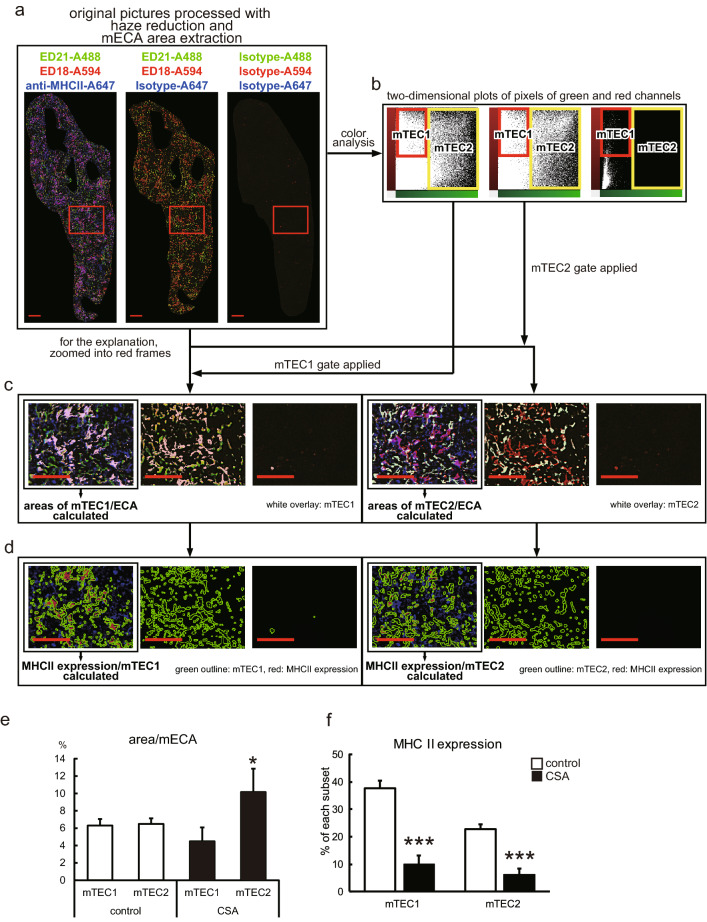
Effects of CSA on the structure and epithelial cells of thymi. **a**–**d** Scheme of the analysis. **a** Sections of thymi were stained with Alexa Fluor 488-conjugated ED21, Alexa Fluor 594-conjugated ED18, and Alexa Fluor 647-conjugated anti-MHCII (OX3) antibodies or corresponding isotype control antibodies. Five randomly selected medullary portions in each sample were captured with BZ-9000. A 20 × objective lens was used. Pictures were combined to recreate the whole medulla. Haze was removed, and mECAs were extracted from each combined picture by BZ-X analyzer software. **b** ED18 single-positive pixels and ED21 single-positive ~ ED18^+^ED21^+^ double-positive pixels were gated as mTEC1 and mTEC2 respectively. **c** The gates were applied to the original pictures. Ratios of the gated mTEC1 or mTEC2 areas against mECA areas were calculated and displayed in (**e**). **d** MHCII positivity in the gated mTEC1 or mTEC2 areas were displayed in (**f**). Each group contains five rats. *p < 0.05, ***p < 0.0005

To clarify the mechanism of reduced development of T cells in CSA-treated thymi, we analyzed thymic sections with multicolor fluorescent immunostaining, and photomicrographs of immunostained sections were taken. Pixels corresponding to the surface areas of mTEC1 and mTEC2 cells were extracted using BZ-X analyzer software (Fig. 3a–c), and MHCII expression by the mTEC subsets (overlap of MHCII on the areas of mTEC subsets) was quantified (Fig. 3d). In reduced medullary areas, a proportion of the mTEC2 area was significantly increased in the CSA-treated thymi (Fig. 3e). In addition, the expression of MHCII molecules was decreased in both subsets (Fig. 3f). The relative increase in mTEC2 and suppression of MHCII expression in both subsets indicate a decrease in mTEC1 subset and suggest that the mTEC population as a whole became less mature and competent.

### Specific tDC subset distribution in mECAs and mEFAs

**Fig. 4:**
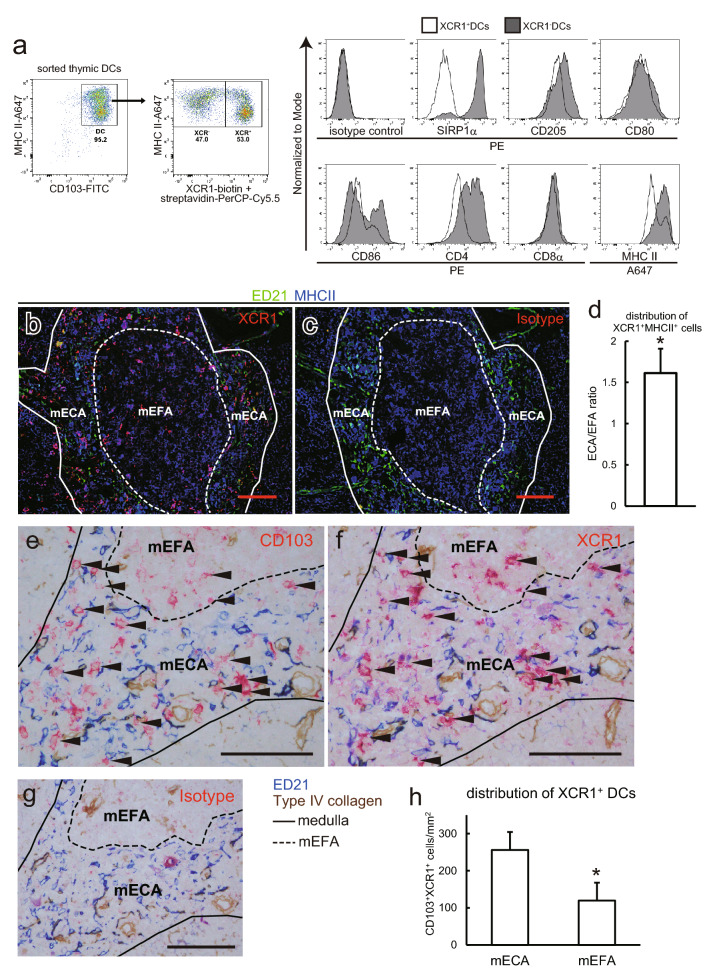
DC subsets in the rat thymus. **a** Low-density CD103^+^ cells were isolated from the thymus of a normal rat and analyzed by flow cytometry. Representative data from four independent analyses are shown. **b**–**d** Thymic sections from control rats were stained with anti-XCR1 (**b**) or mouse IgG2b isotype control (**c**) antibodies followed by biotin-conjugated anti-mouse IgG secondary antibody and Alexa Fluor 594-conjugated streptavidin. After blocking with polyclonal mouse IgG, Alexa Fluor 488-conjugated ED21 and Alexa Fluor 647-conjugated anti-MHCII antibody was applied. Five randomly selected medullary portions in each sample were captured with BZ-9000. A 20 × objective lens was used. Distribution of XCR1^+^MHCII^+^ pixels was analyzed and calculated (**d**). Each group contains five rats. One sample *t* test against value “1” was performed. *p < 0.05. **e**–**h** Sections of normal rat thymi were stained with anti-CD103 (**e**), anti-XCR (**f**), or polyclonal mouse IgG isotype control (**g**) antibodies followed by biotin-conjugated anti-mouse IgG secondary antibody and alkaline phosphatase-conjugated anti-biotin antibody, and then colored with New Fuchsin. After blocking with polyclonal mouse IgG, the sections were stained with purified ED21 antibody, followed by alkaline phosphatase-conjugated anti-mouse IgM antibody, and then colored with Vector Blue to depict mECAs. Type IV collagen was also stained. e and f were serial sections. Pictures were taken with a 40 × objective lens. Scale bars indicate 100 μm. Arrowheads indicate CD103^+^XCR1^+^ cells (XCR1^+^ tDCs). The distribution of XCR1^+^ DCs was calculated and displayed (**h**). Three randomly selected medullas from each rat were analyzed. Three rats were included in this analysis. *p < 0.05. Scale bars in this figure indicate 100 μm. Medullas and mEFAs are indicated by solid and broken lines, respectively

Recently, all conventional DCs in the mouse were reported to be universally subdivided into either XCR1^+^signal regulatory protein 1 α (SIRP1α, CD172a)-negative CD8α^+^ DCs or XCR1^−^SIRP1α^+^CD8α^−^ DCs, regardless of their activation status (Klein et al. 2014; Hasegawa and Matsumoto 2018). We recently reported that rat splenic DCs can also be divided into two populations with several differences from mouse: XCR1^+^SIRP1α^−^CD4^−^CD8α^−^cells and XCR1^−^SIRP1α^+^CD4^+^CD8α^−^ cells (Kitazawa et al. 2019). Although both subsets were CD8α^−^, the XCR1^+^ subset was considered to be the rat counterpart of mouse CD8α^+^ DCs. Accordingly, we analyzed rat tDCs, defined as CD103^+^MHCII^+^ cells (Kitazawa et al. 2019) in flow cytometry, and confirmed the presence of two subsets, XCR1^+^SIRP1α^−^CD4^−^ and XCR1^−^SIRP1α^+^CD4^+^ DCs (Fig. 4a). XCR1^−^ DCs expressed relatively higher levels of CD205 and MHCII than XCR1^+^ DCs.

To examine the localization of tDC subsets in the thymus, we performed multicolor fluorescent immunohistology of thymic sections considering XCR1^+^MHCII^+^ cells as XCR1^+^ DCs (Fig. 4b–d). XCR1^+^MHCII^+^ cells seemed to accumulate in mECAs rather than mEFAs (Fig. 4b). This was confirmed with image analysis (Fig. 4d). For further confirmation, we deployed immunohistology of serial sections to detect CD103^+^XCR1^+^ cells as XCR1^+^ DCs (Fig. 4e–h). CD103-stained (Fig. 4e) and XCR1-stained (Fig. 4f) serial sections were carefully compared to identify CD103^+^XCR1^+^ cells and the distribution was calculated (Fig. 4h), then the accumulation of XCR1^+^ tDCs into mECAs was proven.

Unfortunately, we could not examine the distribution of XCR1^−^ DCs. Although they are SIRP1α^+^, it is difficult to exploit it as a marker because many macrophages express it (Damoiseaux et al. 1989; Hashimoto et al. 2011). Actually, most of SIRP1α^+^ cells were macrophage marker (CD68, CD163, CD169) positive on immunohistology, and an attempt to identify CD103^+^SIRP1α^+^and macrophage marker negative cells on serial sections was unreliable (data not shown).

### Impairment of XCR1^+^ tDCs in the CSA-treated thymus

**Fig. 5:**
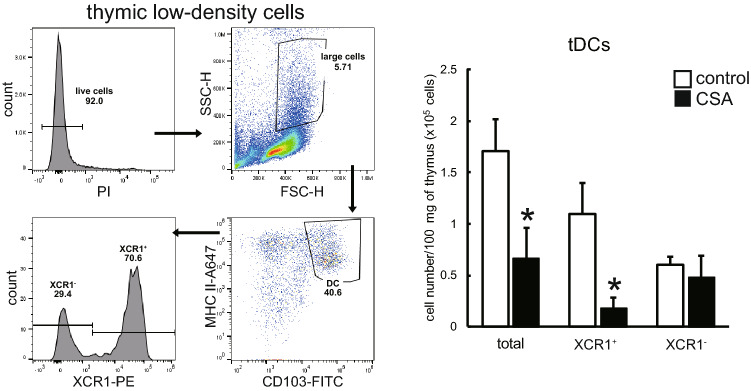
Effects of CSA on thymic dendritic cell (tDC) subsets. Thymic low-density cells from control and CSA-administered rats were subjected to the flow cytometric analysis. The number of tDCs was calculated as [the number of obtained low-density cells × ratio of PI-negative live cells × ratio of large cells on a FSC-SSC plot × ratio of CD103^+^MHCII^+^ cells]. The numbers of XCR1^+^ and XCR1^−^ subsets were calculated within tDCs. Each group contains three rats. *p < 0.05

To investigate the effects of CSA on tDCs, the number of tDCs in CSA-administered rats was calculated as the number of low-density cells obtained from gravity separation multiplied by the ratio of live CD103^+^MHCII^+^ cells. Total tDCs decreased significantly in CSA-administered thymi (Fig. 5), as the XCR1^+^ subset exhibited selective depletion, whereas the XCR1^−^ subset did not change.

### Treg accumulation in mEFAs and effects of CSA on Tregs.

**Fig. 6:**
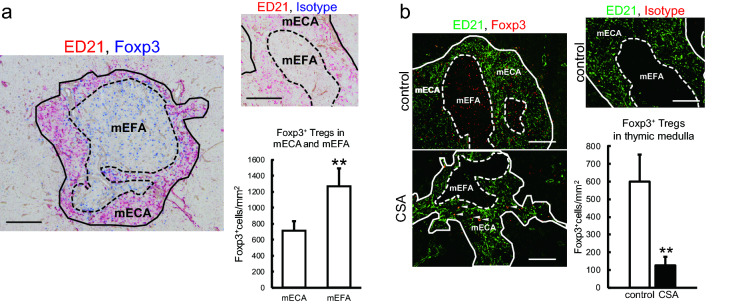
Thymic tTreg localization. **a** Thymic sections from five control rats were stained with biotin-conjugated anti-Foxp3 or biotin-conjugated rat IgG2a isotype control antibodies, followed by alkaline phosphatase-conjugated anti-biotin antibody, and then colored with Vector Blue. Next, the sections were stained with purified ED21 antibody, followed by alkaline phosphatase-conjugated anti-mouse IgM antibody, and then colored with New Fuchsin. Type IV collagen was also stained. Photomicrographs of medullas were taken with a 10 × objective lens. Five medullary portions were randomly selected from each section and the numbers of Foxp3^+^ cells in mECAs and mEFAs (if present) were counted. Scale bars indicate 200 μm. **b** Thymic sections from control and CSA-administered rats were stained with Alexa Fluor 488-conjugated ED21 antibody and biotin-conjugated anti-Foxp3 or rat IgG2a isotype control antibody, followed by Alexa Fluor 594-streptavidin. Photomicrographs of medullas were taken with a 10 × objective lens. Five medullas were randomly selected from each section and the numbers of Foxp3^+^ cells in the medullary areas counted. Nonspecific staining is marked on the image by an arrowhead. Scale bars indicate 100 μm. In this figure, medullas and mEFAs are indicated by solid and broken lines, respectively. Each group contains five rats. **p < 0.005

To identify Tregs by immunohistology, we utilized Foxp3 as a marker. A majority of Foxp3^+^ cells in the thymi were CD4^+^CD25^+^ and tTreg marker Helios^+^ (Thornton et al. 2010) (Fig. S4). In the thymi of control rats, Foxp3^+^ Tregs significantly accumulated in mEFAs compared to mECAs (Fig. 6a). In CSA rats, the total number of Foxp3^+^ Tregs and distribution of Foxp3^+^ Tregs in the thymic medullas were greatly reduced, suggesting impaired generation of tTregs (Fig. 6b).

## Discussion

The present study has several novel findings. First, we found that the proportion of RTEs were greatly reduced. We also observed that CD25^−^ conventional T cells in CSA-administered rats contained significantly more autoreactive cells than control rats. Second, with CSA treatment, the involuted thymic medulla presented a stronger decrease in the mEFA. The fluorescent image analysis revealed that mTECs had a relative decrease in the mTEC1 subset, which has a competent phenotype, as well as downregulation of MHCII molecules in both mTEC1 and mTEC2. Third, in control rats, we observed the presence of two DC subsets equivalent to mouse conventional DCs, XCR1^+^SIRP1α^−^CD4^−^ and XCR1^−^SIRP1α^+^CD4^+^ cells. XCR1^+^ subsets in the medulla had a predominant localization in the previously reported mECAs. On the other hand, the mEFAs contained significantly more Helios^+^Foxp3^+^ tTregs than the ECAs. Finally, with CSA treatment, the XCR1^+^ tDC subset exhibited a selective depletion. Immunohistologically, the total number and distribution of tTregs in the thymic medullas were greatly reduced.

The relative decrease in RTEs defined as CD90^+^ T cells (Hosseinzadeh and Goldschneider 1993) even within decreased peripheral CD4 and CD8 T cells (Fig. 1a) has not yet been reported because no specific markers are available in mice (Fink and Hendricks 2011). This suggests an impaired supply of newly developed T cells from the thymus or higher sensitivity of RTE to CSA than peripheral T cells. Strong reduction of both single-positive thymocytes and mature mTECs as discussed below supports the first possibility.

The major effects of CSA are inhibition of the calcineurin and mitogen-activated protein kinase (MAPK) pathways, both of which are involved in signaling pathways under TCR (Barbarino et al. 2013). Although some publications indicate that MAPK pathways are also involved in the functions of TECs (Colombara et al. 2005; Mainiero et al. 2003; Ramarli et al. 1998), there is no direct evidence that medullary involution and impaired differentiation of mTECs are caused by direct effects of CSA against TECs. On the other hand, signals from the T cell receptor are essential for positive selection (Klein et al. 2014). Moreover, in in vitro experiments, CSA has been shown to inhibit positive selection (Anderson et al. 1995). In turn, although TECs induce the development and selection of thymocytes, they also require molecular interactions with, for example, RANK-RANKL, CD40-CD40L, and lymphotoxin-LTβR for maturation and survival (Alexandropoulos and Danzl 2012). We speculate that CSA affects positive selection first, then it causes the impaired influx of single-positive thymocytes into the medulla, inducing a decrease in medullary areas (Fig. 2i). Eventually, mTECs cannot mature due to a lack of interaction with single-positive thymocytes (Fig. 3e).

The selective accumulation of tTregs in the mEFAs in control rats suggests the presence of a specific domain for the Treg induction in the rat thymus (Fig. 6a), which has not been reported in mice. The reduction of mEFAs (Fig. 2i, j) and depletion of tTregs to one-sixth (Fig. 6b) by CSA administration suggest that the microenvironment of mEFAs loses its function for the Treg induction. However, we could not find an accumulation of particular cells that are responsible for Treg development into these areas. The actual significance of mEFAs is still left to be revealed.

Concerning XCR1^+^tDCs distribution, as XCR1 ligand XCL1 is secreted by mTECs in an AIRE-dependent manner (Lei et al. 2011), it is reasonable that XCR1^+^ tDCs are specifically distributed in mTEC-resident mECAs (Fig. 4d, h). Our finding that XCR1^+^ tDCs decreased in number (Fig. 5) under influence of CSA also reconciles with this XCR1-XCL1 axis concept considering mTEC suppression (Fig. 3e). On the other hand, although we could not examine the distribution of XCR^−^ tDCs, decreased medullary areas and mEFAs may suggest that these tDCs may have lost their original localization and become unable to play their role.

Although we did not perform syngeneic GVHD induction, the simple CSA administration model altered the immunological condition of the animals that can contribute to the onset of the disease. The presence of more autoreactive cells among LN CD25^−^ T cells of CSA rats than control rats indicates a significant increase in the autoreactivity in conventional T cells per se. How was autoreactivity of the T cell pool induced? The increase in the mTEC subset relatively lacking functional molecules and decrease in XCR1^+^ tDCs by CSA treatment suggest impaired negative selection, leading to an increase in autoreactive LN T cells. However, CSA cannot inhibit negative selection directly because it can only partly interfere with some signal pathways during negative selection (DeRyckere et al. 2003). Therefore, it is plausible to speculate that autoreactive TCR can elude negative selection under the effect of CSA via the impaired competency of mTECs. Although it has been reported that a segment Vβ8.5 is abundant among lesion-infiltrating T cells and the periphery of syngeneic GVHD rats (Fischer et al. 1995; Chen et al. 1998), we could not find any skewed Vβ usage when peripheral T cells of CSA rats had increased autoreactivity (data not shown). Autoreactive T cells may be only a small part of Vβ8.5 or other segment positive T cells, and their increase may be undetectable only with Vβ segments. In addition to the enhanced autoreactivity of conventional T cells, the paucity of Tregs should contribute to the onset of syngeneic GVHD.

In this research, CSA induced the impairment of thymic structure, mTEC maturation, tDC localization, conventional T cell generation, Treg generation, and exclusion of autoreactive T cells. After the withdrawal of CSA, the development of autologous GVHD seems to proceed on the balance between these disturbances and recovery from them. Revealing the full view of this phenomenon will give us not only therapeutic medications for autologous GVHD but also more a profound perspective for the understanding of autoimmunity and the treatment of autoimmune diseases and GVHD.

## Supplementary Information

Below is the link to the electronic supplementary material.

Scheme of the mixed lymphocyte reaction. Control and CSA-administered rats were sacrificed and whole/CD25-T cells purified from peripheral lymph nodes, and then stained with CytoTell reagent. These responder T cells were obtained from two rats and pooled. Stimulator DCs were prepared from the spleens of control rats. After 9 days of culture, cells were captured by a flow cytometer and MHCII-PI- live T cells gated, counted, and analyzed for the ratios of proliferating cells. The number of live T cells in each well and the ratios of proliferating (CytoTell low-negative) cells are given in Fig. 1c and d. Each culture was prepared in triplicate. Representative data from two independent experiments were shown (EPS 1574 KB)

Thymic sections of a normal lewis rat (**a**, **b**) or a control rat (**c**–**e**) were stained. The sections were stained with anti-keratin 5 antibody (**a**), biotin-conjugated UEA-1 (**b**), biotin-conjugated ED18 (**c**), biotin-conjugated ED21 (**d**), and biotin-conjugated mouse IgM isotype control antibody (**e**). Anti-keratin 5 antibody was followed by alkaline phosphatase-conjugated anti-rabbit IgG. Biotin-conjugated lectin/antibodies were followed by alkaline phosphatase-conjugated anti-biotin antibody. Photomicrographs were taken with a 20x objective lens. Scale bars indicate 250 μm. Medullas and mEFAs are indicated by solid and broken lines, respectively (EPS 40525 KB)

Immunohistology of the thymic cortex. Thymic sections from control (**a**, **b**) and CSA (**c**, **d**) rats were stained with biotin-conjugated anti-ED19 (**a**, **c**) or purified anti-MHCII (**b**, **d**) antibodies, followed by alkaline phosphatase-conjugated anti-biotin and anti-mouse IgG antibodies, respectively, and colored with Vector Blue substrate. Type IV collagen was also stained. Photomicrographs were taken with a 10x objective lens. Scale bars indicate 100 μm. (EPS 15831 KB)

The majority of Foxp3^+^ thymocytes are CD4^+^CD25^+^Helios^+^ tTregs. The thymus from a normal Lewis rat was digested and analyzed by flow cytometry. The majority of thymic Foxp3^+^ cells were CD4^+^CD25^+^, and more than 85% were Helios^+^. Representative data from four independent analyses are shown (EPS 872 KB)

## Data Availability

No datasets were generated or analyzed during this study. Therefore data sharing is not applicable.
